# Supplementary material for: An ethnographic study of how health system, socio-cultural and individual factors influence uptake of intermittent preventive treatment of malaria in pregnancy with sulfadoxine-pyrimethamine in a Ghanaian context
Source: PLoS One. 2021 Oct 7;16(10):e0257666. doi: 10.1371/journal.pone.0257666 (PMC8496863; doi:10.1371/journal.pone.0257666)
Supplement: Attachment — Submitted filename: Response to Reviewers.docx [file pone.0257666.s006.docx]

Institute of Health Research

University of Health and Allied Sciences

Ho, PMB 31, Volta Region

10^th^ August, 2021

The Editor,

Plos ONE Journal

Dear Editor,

**Response to Ref:** **PONE-D-20-36566R1 “An ethnographic study of how health system, socio-cultural and individual factors influence uptake of intermittent preventive treatment of malaria in pregnancy with sulfadoxine-pyrimethamine in a Ghanaian context”**

Thank you very much for your comments on the above-named manuscript that was submitted to your esteemed journal for consideration. The team has responded to the reviewers’ comments, point by point. They have been uploaded accordingly. Also, the revised manuscript with track changes and a clean copy have been included and the supporting documents as well.

We look forward to hearing favorably form you soon.

Thank you.

Yours truly,

**Dr. Matilda Aberese-Ako (Corresponding author)**

**Response to reviewers**

**Reviewer #1:**

**Many thanks for your kind support. We are grateful. Please find below, a point by point response to your comment.**

Reviewer #1: The authors have done a thorough job of addressing all reviewer comments and I recommend that this paper is accepted. My only minor point of note is to have one last check for typos in the new text (I noticed some in the track changes version but these may have been corrected in the clean version)

**Response: Thank you very much for your feedback and for excellent input. The entire manuscript has been read carefully and revised to correct all typos.**

**Reviewer #2:**

**Many thanks for your kind support. We are grateful. Please find below, a point by point response to your comment.**

Reviewer #2: I read with great interest the revision of this manuscript.

The inclusion of headings and subheadings make the results section clearer to the reader, and facilitates the understanding of the analytical categories identified and used by the researchers. I would only recommend to simplify subheadings, by using more synthetic concepts (e.g. the one used in lines 574-576 is too long).

**Response: Thank you very much for the excellent comment. Several of the headings have been revised to simplify them by using synthetic concepts. Please see lines 307, 391, 436, 574-576, 580 and 674.**

The discussion section shows a deeper interpretation of the results, and includes more complex dialogues with previous studies, with makes the manuscript analytically richer.

Corrections (such the year of the WHO recommendation, and its adaptation in Ghana) and specifications on settings identifiers and data collection techniques have also been introduced.

**Response: Many thanks, we are grateful for your time and insights, which has helped to improve the manuscript**
